# Influence of Glycerol Concentration on the Dissolution Mechanism of Calcium in Water

**DOI:** 10.1021/acs.jpcb.6c01272

**Published:** 2026-05-14

**Authors:** Tatsuya Sasaki, Hidetaka Yamada, Ayaka Suzuki, Takeshi Toyama

**Affiliations:** † Materials Research Laboratory, Technical Development Group, Kobe Steel, Ltd., Kobe, Hyogo 651-2271, Japan; ‡ Frontier Science and Social Co-creation Initiative, 12858 Kanazawa University, Kanazawa, Ishikawa 920-1192, Japan; § Department of Materials and Applied Chemistry, Graduate School of Science and Technology, 12976 Nihon University, Tokyo 101-8308, Japan; ∥ Department of Materials and Applied Chemistry, College of Science and Technology, Nihon University, Tokyo 101-8308, Japan

## Abstract

The structural characteristics of dissolved calcium species in glycerol–water solutions were investigated, and the mechanism by which calcium solubility increased upon increasing the glycerol concentration was elucidated. Comparative dissolution experiments using CaO in various alcohol–water solutions with varying numbers of hydroxyl groups revealed that the calcium solubility increased with increasing number of hydroxyl groups; glycerol and erythritol promoted calcium dissolution to a significantly greater extent than methanol. Structural analysis confirmed the formation of calcium–glycerol complexes and the presence of hydrated species. Evaluation of the solution viscosity and calcium concentration as a function of the glycerol mole fraction identified a critical molar fraction (*X*_glyc_ ≈0.18) at which the hydrogen-bonding network of the solution structure underwent a pronounced transition. Chemical shifts observed via proton nuclear magnetic resonance spectroscopy and spin–lattice relaxation measurements suggested that increasing the glycerol content altered the local water arrangement and enhanced the stability of the calcium–glycerol complexes. At *X*_glyc_ >0.18, the calcium solubility increased sharply due to the preferential formation of stable glycerol-coordinated complexes. These findings establish a critical glycerol concentration threshold for efficient calcium dissolution and offer a new solvent design principle for selective calcium extraction and high-purity CaCO_3_ synthesis.

## Graphical abstract

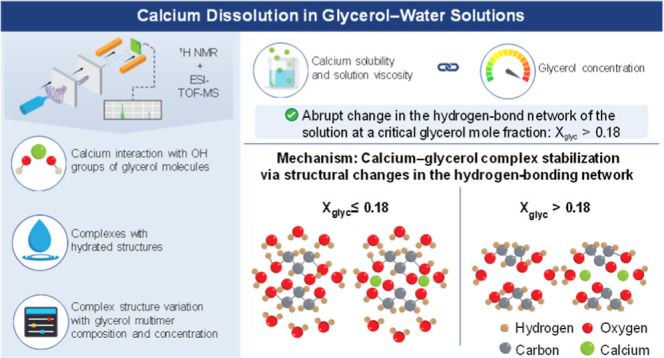

## Introduction

Recently, there has been growing global interest in reducing greenhouse gas emissions as part of climate change mitigation efforts, a key priority aligned with the United Nations Sustainable Development Goals (SDGs).^1^ Among industrialized nations, Japan faces the most significant challenges owing to its heavy reliance on fossil fuels. In 2023, carbon dioxide was the largest contributor to Japan’s greenhouse gas emissions, accounting for ∼0.99 billion tons.^2^ As a key strategy for reducing CO_2_ emissions, technologies for CO_2_ capture, utilization, and storage (CCUS) are being actively developed.^3^ To achieve carbon neutrality by 2050, CO_2_ mineralization shows particular promise as a CO_2_ fixation technology as it does not require hydrogen.

Since alkaline earth metal ions (e.g., Mg^2+^ and Ca^2+^) can react with carbonate ions (CO_3_^2–^) and form stable carbonate species, mineral carbonation using these ions has attracted considerable attention as a CO_2_ sequestration method. Additionally, calcium carbonate (CaCO_3_) has been produced by the reaction of CO_2_ with calcium-containing industrial byproducts, such as waste concrete,^4,5^ concrete sludge,^6^ incineration ash,^7–10^ and steelmaking slag.^4,10–15^ Calcium carbonate is broadly classified into ground calcium carbonate (GCC), a fine powder obtained by physically grinding natural limestone, and precipitated calcium carbonate (PCC), which is chemically synthesized. At present, CaCO_3_ is widely used in the paper, plastic, rubber, and paint industries,^16^ which demand high-whiteness and low-impurity content, thereby rendering synthetic CaCO_3_ a crucial material for meeting these standards. However, obtaining high-purity CaCO_3_ requires the removal of impurities from the calcium source along with the selective extraction of calcium. In this context, steelmaking slag contains highly reactive free CaO (unreacted lime), which can be readily extracted as Ca^2+^ using water or organic solvents, thereby rendering it a useful source of calcium. Thus, the leaching behavior of free CaO plays a crucial role in CaCO_3_ synthesis from steelmaking slag. Specifically, the extraction of free CaO from steelmaking slag using ethylene glycol as a solvent is well-established, and this method can reportedly enable selective calcium leaching.^17,18^ Bubbling CO_2_ through the resulting calcium-containing ethylene glycol solution yields CaCO_3_ as a precipitate.^19^ However, ethylene glycol is both toxic and flammable, and its calcium-dissolving capacity is limited. To address this issue, our group previously identified and evaluated glycerol–water solutions as safer solvents with higher calcium dissolution capacities. For example, it was demonstrated that bubbling CO_2_ gas into a glycerol–water solution containing Ca^2+^ extracted from steel slag generated CaCO_3_.^20,21^ The influence of alkaline conditions on CaCO_3_ formation was also investigated in calcium-containing glycerol–water solutions using Ca­(OH)_2_ as a substitute for the free CaO in the slag.^22^

The physical properties and microstructures of glycerol–water solutions have been widely studied using various techniques, including molecular dynamics simulations,^23–25^ thermodynamic measurements,^26–28^ broadband dielectric spectroscopy,^29,30^ nuclear magnetic resonance (NMR) spectroscopy,^31–33^ infrared spectroscopy,^34,35^ and Raman spectroscopy.^36^ Additionally, the solvation structure of calcium in alcoholic solvents (e.g., methanol, ethanol, and their aqueous solutions) has been the subject of extensive research.^37–40^

However, the properties and structures of systems in which salts (such as calcium) coexist in glycerol–water solutions are largely unknown. Consequently, the mechanism responsible for the high calcium dissolution capacity of glycerol–water mixtures, as well as the influence of the glycerol/water ratio on calcium solubility, remain poorly understood. Thus, to achieve highly efficient CaCO_3_ synthesis for CO_2_ fixation, the effects of different alcohol species and concentrations on the calcium dissolution mechanism must be clarified.

With these considerations in mind, the current study focuses on the calcium dissolution capacity of alcohol–water solutions containing alcohols with different numbers of hydroxyl (OH) groups. Additionally, the structures of the calcium-containing glycerol–water solutions are analyzed using time-of-flight mass spectrometry (TOF-MS) and NMR spectroscopy to elucidate the mechanism of calcium dissolution.

## Experimental Methods

### Effect of the Alcohol on Calcium Dissolution

A range of alcohol–water mixtures were investigated as potential solvents for calcium dissolution, namely, methanol (special grade, Kanto Chemical Co., Tokyo, Japan), ethylene glycol (special grade, Kanto Chemical Co., Tokyo, Japan), glycerol (special grade, Kanto Chemical Co., Tokyo, Japan), and erythritol (>99.0%, Tokyo Chemical Industry Co., Tokyo, Japan). Ca­(OH)_2_ (special grade, 99.9% purity, Kanto Chemical Co., Tokyo, Japan) was used as the calcium source. Due to the increased viscosity and poor filterability upon the dissolution of calcium in the erythritol–water solutions, the mole fraction of alcohol in the water solution was set at 0.083 in all systems.

An aliquot (0.1 L) of the alcohol–water solution was placed in a 0.2 L beaker, and Ca­(OH)_2_ was added to yield a suspension containing 135 mM Ca­(OH)_2_. The resulting mixture was stirred using a magnetic stirrer at 25 °C for 60 min to dissolve the Ca­(OH)_2_. Subsequently, the suspension was filtered through a glass fiber filter (retention 0.3 μm, GF-75, ADVANTEC TOYO KAISHA, Ltd., Tokyo, Japan) to obtain the calcium-containing alcohol solution. The Ca^2+^ concentration in the filtrate was measured by inductively coupled plasma–optical emission spectrometry (ICP-OES; iCAP 7400 Duo MFC, Thermo Fisher Scientific Inc., Massachusetts, USA). The calcium/alcohol molar ratio varied slightly depending on the type of alcohol but was approximately within the range of 1:6–1:8.

### Evaluation of the Dissolution Structure

To investigate the dissolution structure of Ca in glycerol, solvents were prepared by mixing glycerol with either H_2_O (resistivity at 25 °C > 15.0 MΩ cm, Milli-Q IQ 7000 type-LC, Merck KGaA, Darmstadt, Germany) or D_2_O (>99.8% D, Fujifilm Wako Pure Chemical Corp., Osaka, Japan). Glycerol (special grade, Fujifilm Wako Pure Chemical Corp., Osaka, Japan) was used as received, and CaO was employed as the calcium source (special grade, 99.9%, Fujifilm Wako Pure Chemical Corp., Osaka, Japan). The glycerol–water mixtures were prepared using glycerol molar fractions (*X*_glyc_) ranging from 0.095 to 0.27.

Each glycerol–water solution was placed in a polyethylene container, and excess CaO was added. Following a previous report,^22^ the obtained mixture was stirred with a magnetic stirrer at 25 °C for 60 min to give a saturated solution, as indicated by the presence of some undissolved CaO at the bottom of the container. Subsequent filtration through a glass fiber filter (pore size 1.0 μm, Advanced Microdevices Pvt. Ltd., Ambala, India) yielded the calcium-containing glycerol solution.

For the solutions prepared using heavy water, ^1^H NMR spectra were recorded using a VNMRS 600 MHz NMR spectrometer (Agilent Technologies Inc., California, USA). For samples prepared using light water, the dissolved calcium species were analyzed by electrospray ionization TOF-MS (ESI-TOF-MS, MS Compact, Bruker Co., Massachusetts, USA) using a direct infusion method. In each case, a control sample (CaO-free glycerol–water solution) was prepared and analyzed for comparison under the same conditions. To perform the NMR measurements, each sample was sealed in an NMR capillary tube (N-502B, Nihon Seimitsu Kagaku Co. Tokyo, Japan) and placed inside a standard 5 mm NMR tube (N-5PW, Nihon Seimitsu Kagaku Co. Tokyo, Japan). The chemical shifts were referenced to an external standard, namely, a D_2_O solution of TSP (99.9 at % D, containing 0.05 wt % 3-(trimethylsilyl)­propionic-2,2,3,3-*d*_4_ acid sodium salt, Merck KGaA, Darmstadt, Germany). The spectrometer was shimmed such that the resonance of the TSP protons appeared at 0 ppm.

### Evaluation of the Viscosity and Ca Dissolution Amount

Glycerol–water solutions were prepared using *X*_glyc_ values ranging from 0 to 0.27. Each solution was placed in a polyethylene container, a large excess of CaO was added, and the mixture was stirred with a magnetic stirrer at 30 °C for 24 h to dissolve the CaO. During stirring, high-purity N_2_ gas (>99.999% N_2_, Shinko Air Water Gas Co., Osaka, Japan) was bubbled through the suspension at a rate of 0.1 L min^–1^ to prevent absorption of atmospheric CO_2_. After 24 h, some residual undissolved CaO remained at the bottom of the container, confirming that a saturated solution had been prepared. The mixtures were then filtered to obtain clear Ca-saturated glycerol solutions. Specifically, the solutions with *X*_glyc_ ≤0–0.20 were filtered through an ultrahigh-molecular-weight polyethylene filter (pore size 10 nm, Entegris Inc., Massachusetts, USA), and those with *X*_glyc_ >0.20–0.27 were filtered through a Hydrophilic PTFE type membrane filter (pore size 1.0 μm, ADVANTEC TOYO KAISHA, Ltd., Tokyo, Japan). The Ca concentration obtained after 60 min was comparable to that reported in a previous study,^22^ as discussed later, confirming that extending the extraction time to 24 h had no significant effect.

The density and kinematic viscosity of each filtered solution were subsequently measured to determine the dynamic viscosity. Specifically, the density was measured using a vibrating-tube densitometer (DMA 4500, Anton Paar GmbH, Graz, Austria), and the kinematic viscosity was measured using a Canon–Fenske viscometer (Automatic Viscometer VMC-222, for opaque liquids, Rigo Co., Saitama, Japan). The viscosity was calculated from the product of the kinematic viscosity and density. Finally, the Ca^2+^ concentration of each solution was measured by ICP-OES (Agilent 5110 ICP-OES, Agilent Technologies Inc., California, USA).

## Results and Discussion

### Effect of Calcium on the Solution Structure

#### Dissolution Behavior of CaO in the Alcohol–Water Solutions

The solubility of calcium in ethylene glycol–water and glycerol–water solutions has been reported previously,^19^ with glycerol–water exhibiting a higher calcium dissolution capacity than ethylene glycol–water. This difference is attributed to the number of OH groups in each alcohol molecule. Therefore, for the purpose of this study, methanol (one OH group), ethylene glycol (two OH groups), glycerol (three OH groups), and erythritol (four OH groups) were selected as representative alcohol solvents to compare the dissolution behavior of CaO. The results are shown in Figure 1.

**Figure 1:**
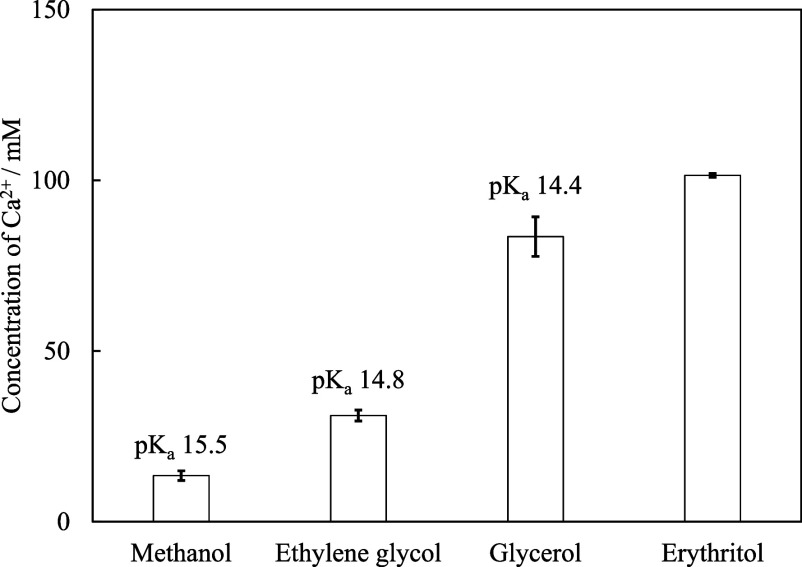
Comparison of the amount of dissolved calcium in various alcohol-based solvents (*X*_alcohol_ = 0.083). *To the best of our knowledge, no p*K*_a_ value has been reported for erythritol.

As indicated in the figure, the calcium concentration in methanol reached only ∼13.5 mM; however, upon increasing the number of OH groups in the solvent molecule, the amount of dissolved calcium increased, reaching ∼101.4 mM in the erythritol–water solution. The relationship between the p*K*_a_ of each alcohol (excluding erythritol, which has no reported p*K*_a_)^41^ and the amount of dissolved calcium was subsequently evaluated. Although glycerol contains two structurally distinct types of OH groups, namely, primary and secondary OH groups, the p*K*_a_ value reported herein represents an experimentally determined value that does not distinguish between these two types of OH groups. Solvents with lower p*K*_a_ values tended to enhance calcium dissolution, supporting the hypothesis that Ca^2+^ dissolution was promoted by a greater number of OH groups. This suggests that the dissolution of calcium in a glycerol–water solution is likely facilitated by interactions with the glycerol OH groups, presumably through complex formation with Ca^2+^.

#### ^1^H NMR Evaluation of the Calcium-Containing Glycerol–Water Solution Structure

Glycerol–water solutions are considered safer solvents and exhibit a higher capacity for calcium dissolution than those prepared using other alcohols. Consequently, subsequent investigations focused exclusively on the glycerol–water system to evaluate their suitability for calcium extraction, although the precise mechanism underlying calcium dissolution in these media is yet to be fully elucidated. ^1^H NMR spectroscopy was employed to investigate the effect of calcium on the glycerol OH groups in solution. Figure 2 shows the ^1^H NMR spectra recorded for a CaO-containing glycerol–water solution (*X*_glyc_ = 0.14) at 20 °C. For comparison, the corresponding spectrum for a glycerol–water solution (*X*_glyc_ = 0.14) containing no CaO is also shown. Upon the addition of calcium, the full width at half-maximum of the NMR signal attributable to the glycerol OH protons became markedly narrower. In general, the line width of the OH proton signal in such systems is broadened by proton exchange with the surrounding water molecules. Therefore, the significant narrowing of the OH proton signal indicated that the rate of proton exchange with water was suppressed. This observation suggests that calcium ions interact with the OH groups of glycerol instead of water, i.e., calcium is dissolved in the solution through coordination with the OH groups of glycerol to form a complex, rather than existing as solvated Ca^2+^ ions in solution.

**Figure 2:**
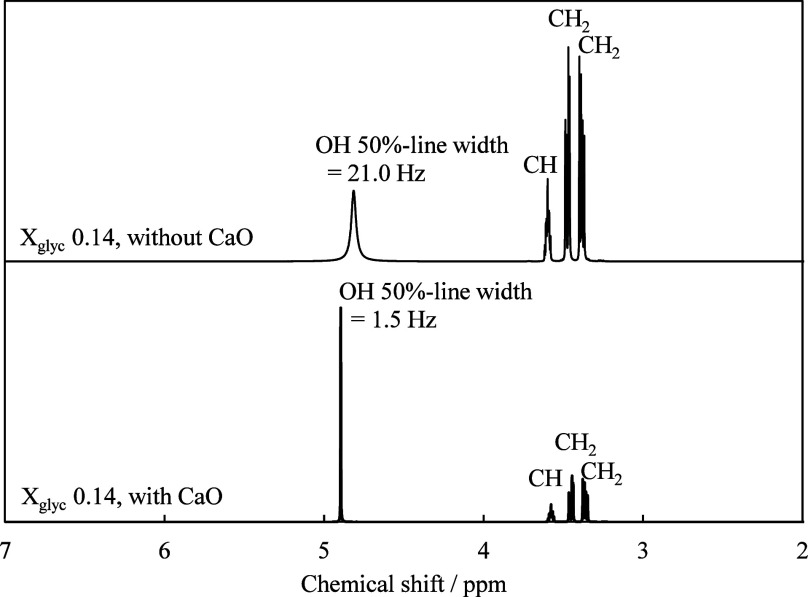
^1^H NMR spectra of the glycerol–water solutions (*X*_glyc_ = 0.14) with and without CaO.

To elucidate the structures of the calcium species formed through coordination with the glycerol OH groups, ESI-TOF-MS was employed to analyze the glycerol–water solutions containing dissolved CaO. Figure 3 shows the mass spectrum recorded for a CaO-containing glycerol–water solution (*X*_glyc_ = 0.14), wherein a peak corresponding to protonated glycerol ([glycerol + H]^+^) can be clearly observed at *m*/*z* 93. Importantly, a peak was detected at *m*/*z* 131, which was assigned to the calcium–glycerol complex ([Ca–glycerol + H]^+^). Additionally, a series of peaks were present at *m*/*z* 149 and 158, increasing in *m*/*z* increments of 9. These peaks can be explained by considering the divalent nature of Ca^2+^; therefore, complexes with a charge of +2 appear at half of their actual mass-to-charge ratio. Specifically, the observed 9 *m*/*z* difference corresponds to a mass difference of 18, consistent with the mass of a water molecule. This series of peaks therefore indicates the presence of calcium–glycerol complexes that differ only in their degree of hydration (i.e., partially hydrated vs hydrated complexes).

**Figure 3:**
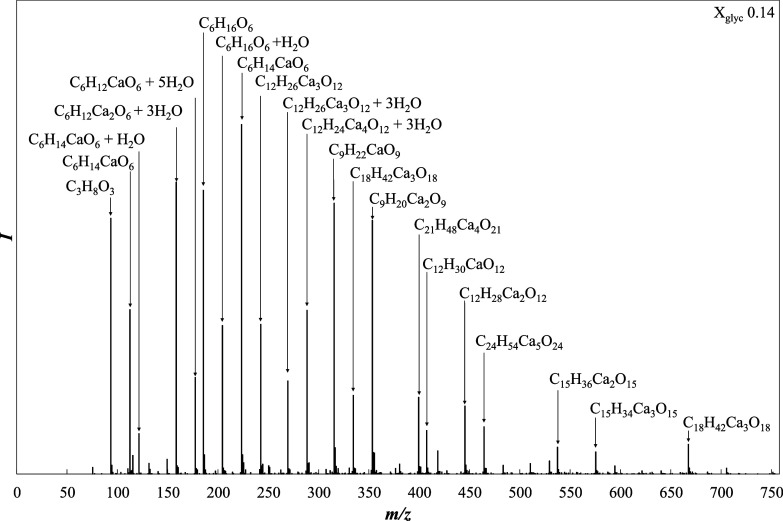
TOF-MS results for a CaO-dissolved glycerol–water solution (*X*_glyc_ = 0.14).

Furthermore, peaks corresponding to species containing multiple glycerol molecules (e.g., glycerol dimers and trimers coordinated to calcium) were observed, confirming the existence of a variety of complex species. This implies that, upon the dissolution of CaO in a glycerol–water solution, Ca^2+^ can associate with one or more glycerol molecules with varying degrees of hydration. The distribution and proportion of these species likely depend on the composition of the solution (glycerol concentration and water content); such variations in chemical speciation are expected to influence the macroscopic solution properties, such as the viscosity, as discussed later.

The possible structures of the calcium–glycerol complexes inferred from these results are shown in Figure 4. Specifically, based on the NMR spectroscopic and MS observations, it appeared that a monomeric complex was formed in solution, wherein Ca^2+^ is chelated by the two terminal OH groups of a single glycerol molecule, likely involving deprotonation of the terminal OH groups (Figure 4, left). A dimeric complex may also exist, in which a single Ca^2+^ ion is sandwiched between and coordinated by two glycerol molecules through the deprotonated terminal OH groups (Figure 4, right). In both cases, Ca^2+^ can coordinate with a few water molecules in its first solvation shell to form a hydrated complex. Notably, the NMR measurements provide an average view of the solution structure and do not directly reveal specific complex structures; however, the combination of NMR and MS evidence supports the above description.

**Figure 4:**
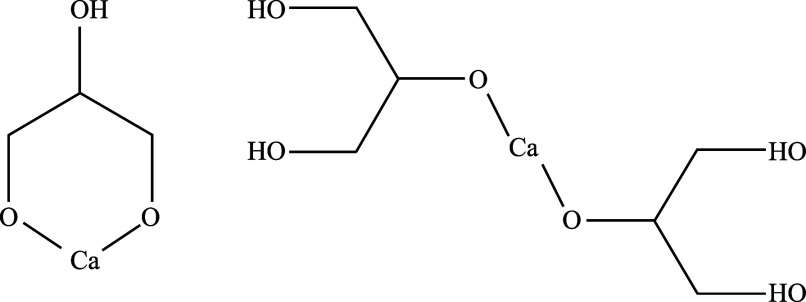
Estimated structures of the calcium–glycerol complexes (left: monomeric complex containing a single glycerol molecule; right: dimeric complex with two glycerol molecules coordinating to a single Ca^2+^ center).

Thus, in the glycerol–water solution, Ca^2+^ dissolves predominantly through interactions with the OH groups of glycerol, likely involving partial deprotonation and the formation of Ca–O coordination bonds. Although the possible formation of hydroxide complexes through interactions between Ca^2+^ and OH^–^ as well as ion-pair formation driven by Coulombic interactions cannot be excluded in this study, such species were not detected in the ESI-TOF-MS measurements. The combined NMR and MS results nevertheless indicate that Ca^2+^ primarily coordinates with the OH groups of glycerol to form complexes, which is considered to be the primary origin of the high calcium solubility observed in the glycerol–water solutions.

### Effect of the Glycerol/Water Ratio on the Calcium Complex Structure in Glycerol–Water Solutions

#### Viscosity and Dissolved Calcium Concentration at Different Glycerol Concentrations

While the above analyses revealed that calcium forms complexes with glycerol in solution, the dispersion or association of these complexes in solution remained unclear. Generally, complex formation in solutions can enhance the intermolecular interactions to increase the solution viscosity. For example, in polymer solutions, higher degrees of macromolecular association lead to higher viscosity,^42^ and in silicate melts, more extensive Si–O–Si polymerization is correlated with increased viscosity.^43^ By analogy, measuring the viscosity of calcium-containing glycerol solutions may provide insights into the degree of complex associations or interactions occurring in the solutions.

The viscosities of glycerol–water solutions prepared using various glycerol concentrations (*X*_glyc_) and containing dissolved CaO were measured at 30 °C and compared with those of CaO-free glycerol solutions of the same composition. As shown in Figure 5, the glycerol solutions containing dissolved CaO exhibited higher viscosity than the corresponding Ca-free solutions, consistent with the presence of calcium–glycerol complexes, which increased the intermolecular forces in solution. Moreover, as the glycerol concentration was increased (i.e., the water content was decreased), the viscosity increased for the Ca-containing solutions. This increase was particularly pronounced when *X*_glyc_ exceeded ∼0.18, beyond which the viscosity increased sharply.

**Figure 5:**
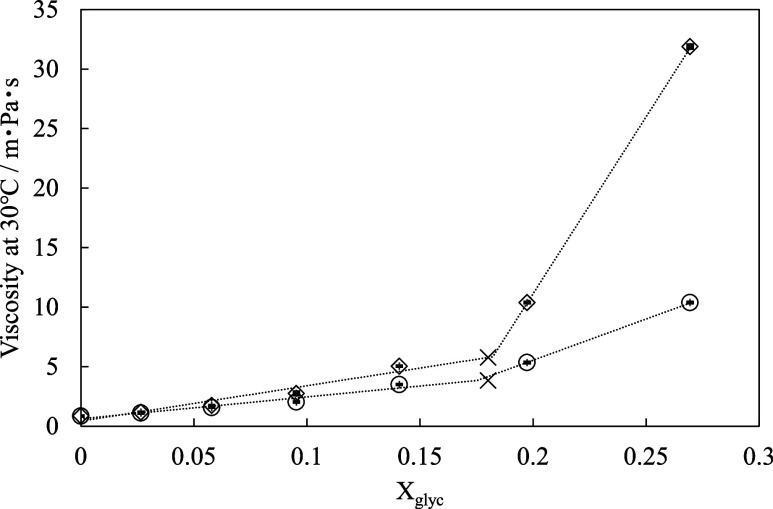
Effect of the water content on the viscosity of glycerol–water solutions at 30 °C. ◊: with CaO; ○: without CaO, ×: threshold.

As shown in Figure 6, the maximum amount of Ca^2+^ that could be dissolved in the glycerol–water mixture increased with the glycerol fraction. In particular, as *X*_glyc_ increased, especially beyond ∼0.18, the dissolved calcium concentration exhibited a sharp exponential increase. This suggests that a higher glycerol content promotes the formation and stabilization of calcium–glycerol complexes, thereby enabling more calcium to remain in solution. Notably, the abrupt increase in viscosity observed around this glycerol concentration coincides with the composition at which the calcium solubility also increases dramatically. This suggests that a significant structural change occurs in the solution near the critical glycerol concentration, which is likely related to the interaction or aggregation of calcium complexes when the water fraction becomes sufficiently low.

**Figure 6:**
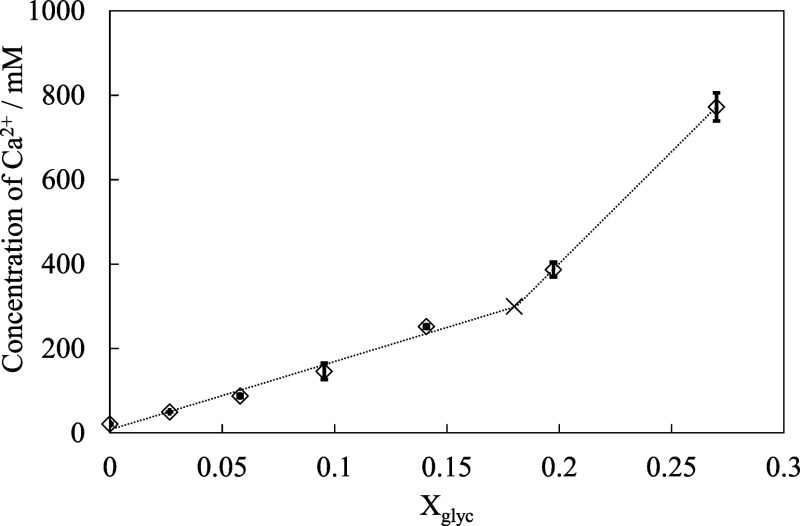
Effect of the water content on the dissolved calcium concentration in glycerol–water solutions at 30 °C (saturated with CaO). ×: threshold.

To quantitatively determine this threshold glycerol concentration, the data in Figures 5 and 6 were analyzed using a continuous two-segment piecewise linear regression model (eq 1). The transition concentration was treated as an unknown parameter and determined by minimizing the residual sum of squares via a grid-search approach. The 95% confidence interval of the transition concentration was estimated by residual bootstrap analysis, and the results are summarized in Table 1. The estimated transition concentration was *X*_glyc_ = 0.18 for the viscosity of Ca-free solutions (Figure 5) and *X*_glyc_ = 0.18 for the viscosity of Ca-containing solutions (Figure 6), with 95% confidence intervals ranging from 0.16 to 0.19 in both cases. The fitted regression results are shown as dashed lines in Figures 5 and 6, and the estimated parameters for each linear segment are summarized in Table 1. The obtained model reproduces the experimental data with residual errors comparable to those of conventional segmented regression models, while providing a statistically defined threshold glycerol concentration. Since the threshold concentrations independently determined from Figures 5 and 6 are in close agreement, these results support the existence of a critical glycerol concentration. This behavior suggests that a significant structural change occurs in the solution near this concentration, which is likely associated with enhanced interactions or aggregation of calcium complexes under sufficiently low water fraction conditions.

1 $$y\left(x\right)=a+bx+d{\left(x-c\right)}_{+},{\left(x-c\right)}_{+}=\{\begin{array}{ll} 0, & \left(x\leq c\right)\\ x-c, & \left(x>c\right) \end{array}$$

**Table 1:** Threshold Glycerol Concentrations and Bootstrap Confidence Intervals Obtained from Segmented Linear Regression Analyses of the Data in Figures 5 and 6 ^a^

	threshold	95% confidence interval (bootstrap)	SSE
viscosity without CaO (Figure 5)	0.18	0.16, 0.19	0.25
viscosity with CaO (Figure 5)	0.18	0.18, 0.19	0.75
calcium concentration (Figure 6)	0.18	0.17, 0.19	907.37

a SSE; sum of squared errors

#### ^1^H NMR Spectroscopic Evaluation of Calcium-Containing Glycerol–Water Solutions of Different Concentrations

To clarify the influence of the glycerol–water ratio on the local structures of the calcium–glycerol complexes, the chemical shifts of the CH (methine/methylene) proton signals were observed in the ^1^H NMR spectra for solutions with different values of *X*_glyc_. Figure 7 shows the chemical shifts of the glycerol CH protons as a function of glycerol concentration for solutions with and without CaO. As mentioned above, the OH proton signals were not used for this comparison because any subtle shifts due to complex formation with Ca were obscured by exchange with the large excess of water.

**Figure 7:**
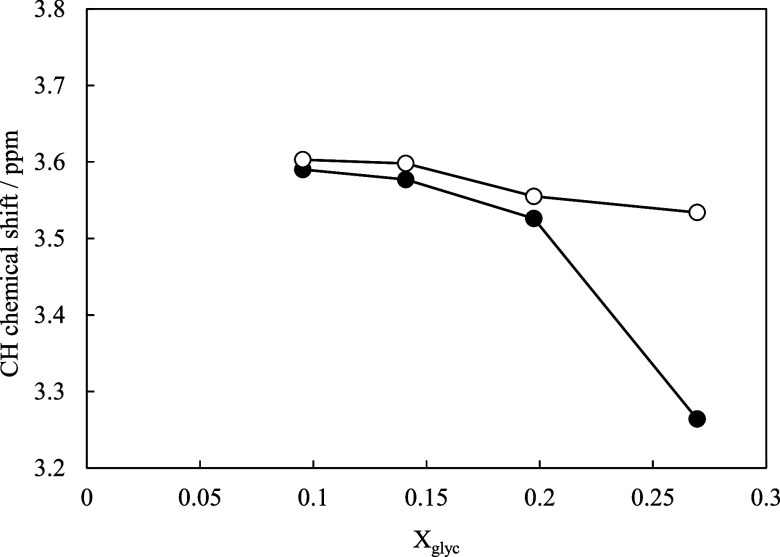
Chemical shifts of the glycerol CH proton signals in the ^1^H NMR spectra for solutions with different values of *X*_glyc_. ●: with CaO; ○: without CaO.

In CaO-free glycerol–water solutions, as X_glyc_ was increased, the resonance of the glycerol CH protons shifted to a higher field (i.e., a lower chemical shift). This observation is consistent with a report by Dashnau et al.,^25^ which noted that as the glycerol content in a glycerol–water mixture increased, the number of H_2_O molecules in the vicinity of the glycerol CH groups diminished. In the current study, the addition of CaO caused the CH proton signal to shift further upfield compared with the corresponding signal of the Ca-free solution at the same *X*_glyc_ value. This can be accounted for by considering that calcium possesses low electronegativity (∼1.0), and upon complex formation with glycerol, it likely coordinates with nearby water molecules in its hydration shell. Consequently, even fewer water molecules remain in the immediate environment of the glycerol CH group. The more pronounced chemical shift observed in the Ca-containing solutions can therefore be explained by the reduced number of local water molecules around the CH protons due to calcium complexation. This finding is in good agreement with the viscosity behavior (Figure 5), which indicates that increasing the glycerol content and the incorporation of calcium lead to a structural environment where glycerol molecules experience fewer interactions with water.

Additionally, changes in the calcium concentration were accompanied by variations in the relative abundance of OH^–^ ions in solution, leading to an increase in pH, which can also influence the chemical shifts and relaxation times (*T*_1_) observed during NMR measurements. In the Ca^2+^-free system, the upfield shift detected for the CH proton signals with increasing *X*_glyc_ was considered to reflect changes in the local polarity associated with the increasing glycerol concentration. In contrast, in the presence of Ca^2+^, the CH proton signals shifted further upfield even at the same *X*_glyc_, and a concomitant decrease in *T*_1_ was observed. These changes were considered to arise primarily from the preferential interactions of Ca^2+^ with the OH groups of glycerol rather than with water molecules, leading to modifications in the local hydrogen-bonding network and the arrangement of surrounding water molecules. Accordingly, the observed variations in the NMR parameters were likely caused by both Ca^2+^-induced changes in the chemical equilibria and pH, and local structural modifications associated with calcium–glycerol complex formation.

The interactions between the glycerol molecules, calcium, and water were further probed by measuring the spin–lattice relaxation time (*T*_1_) of the glycerol protons in these solutions. Figure 8 summarizes the *T*_1_ values obtained for the glycerol OH protons (left) and CH protons (right) as a function of *X*_glyc_ for solutions both with and without CaO. In the case of the CaO-free glycerol–water solutions, the *T*_1_ values of both the OH and CH protons decreased with increasing glycerol concentration. Notably, a shorter *T*_1_ generally indicates that the local environment of the proton facilitates more efficient relaxation, often due to restricted molecular mobility or enhanced dipole–dipole interactions. The decrease in *T*_1_ with increasing *X*_glyc_ therefore suggests that the OH and CH protons of glycerol experience additional frictions or interactions at higher glycerol concentrations. In particular, the *T*_1_ of the OH proton decreased sharply with the increasing glycerol content, implying that as the number of water molecules reduced, the glycerol OH groups increasingly participated in hydrogen bonding with either water or other glycerol molecules, thereby restricting their motion.

**Figure 8:**
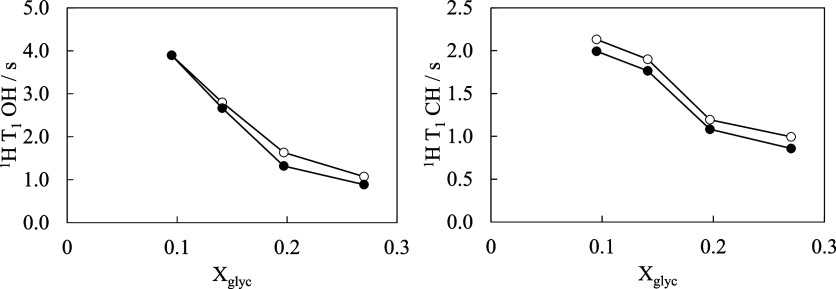
Spin–lattice relaxation times (*T*_1_) of the glycerol protons (measured by ^1^H NMR spectroscopy) as a function of glycerol concentration. Left: *T*_1_ for the glycerol OH protons; Right: *T*_1_ for the glycerol CH protons. ●: with CaO; ○: without CaO.

In the calcium-containing systems, the *T*_1_ values of the OH and CH protons were further reduced compared with the corresponding values in the calcium-free systems. Emilia et al.^38^ investigated the solvation behavior of calcium in methanol and methanol–water solutions using molecular dynamics simulations and reported that in the methanol–water–calcium system, calcium preferentially dissolves in water over methanol. However, in the glycerol–water–calcium system, the multiple OH groups of glycerol allow multisite coordination. This leads to the hypothesis that Ca^2+^ can interact strongly with both water and glycerol, which promotes the formation of a localized solvation structure, which in turn reduces the mobility of the glycerol protons, as reflected by the observed shortening of *T*_1_. Therefore, these NMR spectroscopic results support the idea that the presence of calcium strengthens the hydrogen-bonding network in solution, consistent with the observed increase in viscosity at higher calcium concentrations.

#### Influence of the Glycerol Concentration on the Hydrogen-Bonding Network and the Calcium Complex Stability

In binary Ca-free glycerol–water systems, the glycerol concentration strongly influences the hydrogen-bonding network of water. Specifically, increasing the glycerol content disrupts the bulk water hydrogen-bonding network, reducing its overall extent. In practical terms, when the glycerol concentration becomes sufficiently high, very little “free” bulk water remains; instead, the majority of water molecules occupy the first hydration shell of glycerol. Under these conditions, the dominant arrangement of water shifts, wherein molecules that initially interacted with the hydrophobic CH regions of glycerol become increasingly engaged with its hydrophilic OH groups. Consequently, direct glycerol–glycerol interactions (e.g., between the carbon backbones of neighboring glycerol molecules) are strengthened, leading to increased solution viscosity,^24^ consistent with the observations presented in the preceding sections.

In the ternary glycerol–water–calcium system, the glycerol concentration affected not only the hydrogen-bonding environment but also the stability of the calcium–glycerol complexes due to strong binding between the Ca^2+^ ions and the glycerol OH groups. A schematic representation of the proposed model for the concentration-dependent hydrogen-bonding network is presented in Figure 9. When the glycerol concentration is relatively low (*X*_glyc_ ≤0.18), a substantial amount of free bulk water is present in solution. These free water molecules can interact with calcium–glycerol complexes, effectively perturbing the coordination between Ca^2+^ and glycerol. This interference by water likely destabilizes the complexes, which explains the comparatively limited calcium solubility observed under these conditions (see Figure 6). In contrast, at higher glycerol concentrations (*X*_glyc_ >0.18), the hydrogen-bonding network of the bulk water largely collapsed, and essentially all water was associated with glycerol or calcium. In this regime, the calcium–glycerol complexes encountered far fewer disruptive interactions with water, resulting in greater complex stability. This heightened stability manifests as a dramatically increased calcium dissolution above the critical concentration (i.e., *X*_glyc_ ≈0.18). The extent to which similar trends are observed in the molecular dynamics studies of this investigation or in other alcohol–water solution systems remains a topic for future research.

**Figure 9:**
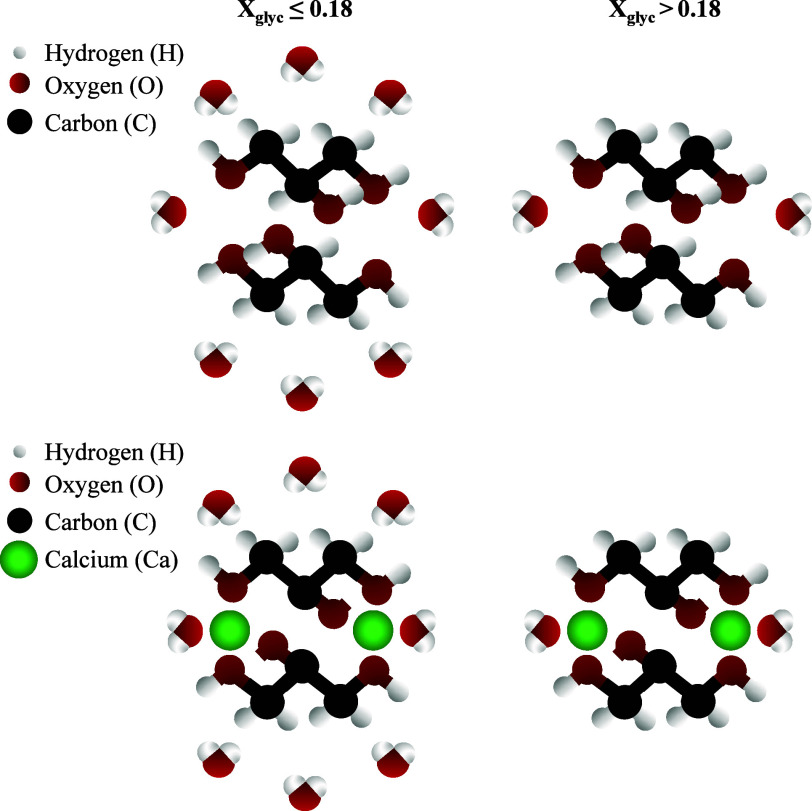
Schematic illustration of the proposed model for the hydrogen-bonding network and indication of the calcium–glycerol complex stability with (lower) and without (upper) Ca^2+^ at a low glycerol concentration (left, *X*_glyc_ ≤0.18) and a high glycerol concentration (right, *X*_glyc_ >0.18).

## Conclusions

This study elucidated the dissolution mechanism of calcium in alcohol–water solutions, focusing particularly on detailed investigations of complex formation behavior and changes in the solvation structure of glycerol–water solutions. It was found that the calcium solubility was strongly dependent on the number of OH groups in the alcohol molecule, increasing with higher OH group content. For glycerol and erythritol, a marked enhancement in calcium solubility was observed, attributable to complex formation between calcium and the OH groups of the alcohol molecules. The results of ^1^H nuclear magnetic resonance spectroscopy and electrospray ionization time-of-flight mass spectrometry suggested that calcium interacts with the glycerol OH groups to form complexes, some of which possess hydrated structures. Furthermore, association with glycerol multimers was confirmed, revealing that the complex structure varies significantly depending on the solution composition and concentration. Both the calcium solubility and the solution viscosity increased at higher glycerol concentrations, with a particularly abrupt change being observed at a glycerol mole fraction of >0.18. This behavior was thought to result from the stabilization of calcium complexes due to structural changes in the hydrogen-bonding network. The findings of this study therefore offer new guidelines for solvent design in CO_2_ fixation methods using calcium extraction and carbonate synthesis in glycerol–water systems, offering essential insights to support the advancement of environmentally compatible CO_2_ capture and utilization strategies. It should be noted, however, that these solution structures were not directly observed. Direct elucidation of the detailed solution structures as well as verification of the associated hydrogen-bonding dynamics will therefore be investigated in future studies. Future research in our group will also focus on the optimization of the carbonate synthesis process, and the results will be reported in due course.

## References

[1] United Nations . Transforming Our World: The 2030 Agenda for Sustainable Development; United Nations: New York, 2015. https://sdgs.un.org/sites/default/files/publications/21252030%20Agenda%20for%20Sustainable%20Development%20web.pdf (accessed 24 Dec, 2025).

[2] Ministry of the Environment, Japan . Japan’s national greenhouse gas emissions and removals in FY2023 <Executive Summary>, 2025. https://www.env.go.jp/content/000310707.pdf (accessed 8 Dec, 2025).

[3] Nakagaki T. (2019). Structure
of anthropogenic CO_2_ emission sources and perspectives
for reduction technologies. Bull. Iron Steel
Inst. Japan.

[4] Iizuka A., Fujii M., Yamasaki A., Yanagisawa Y. (2002). Waste treatment
technologies. A novel reduction process of CO_2_ fixation
by waste concrete treatment. Kagaku Kogaku Ronbunshu.

[5] Kunzler C., Alves N., Pereira E., Nienczewski J., Ligabue R., Einloft S., Dullius J. (2011). CO_2_ storage
with indirect carbonation using industrial waste. Energy Procedia.

[6] Iizuka A., Honma M., Hayakawa Y., Yamasaki A., Yanagisawa Y. (2012). Aqueous mineral
carbonation process via concrete sludge. Kagaku
Kogaku Ronbunshu.

[7] Rendek E., Ducom G., Germain P. (2006). Carbon dioxide
sequestration in municipal
solid waste incinerator (MSWI) bottom ash. J.
Hazard. Mater..

[8] Montes-Hernandez G., Pérez-López R., Renard F., Nieto J. M., Charlet L. (2009). Mineral sequestration
of CO_2_ by aqueous
carbonation of coal combustion fly-ash. J. Hazard.
Mater..

[9] Wang L., Jin Y., Nie Y. (2010). Investigation
of accelerated and natural carbonation
of MSWI fly ash with a high content of Ca. J.
Hazard. Mater..

[10] He L., Yu D., Lv W., Wu J., Xu M. (2013). A novel method for
CO_2_ sequestration via indirect carbonation of coal fly
ash. Ind. Eng. Chem. Res..

[11] Kodama S., Nishimoto T., Yamamoto N., Yogo K., Yamada K. (2008). Development
of a new pH-swing CO_2_ mineralization process with a recyclable
reaction solution. Energy.

[12] Teir S., Eloneva S., Fogelholm C. J., Zevenhoven R. (2007). Dissolution
of steelmaking slags in acetic acid for precipitated calcium carbonate
production. Energy.

[13] Eloneva S., Said A., Fogelholm C. J., Zevenhoven R. (2012). Preliminary
assessment of a method utilizing carbon dioxide and steelmaking slags
to produce precipitated calcium carbonate. Appl.
Energy.

[14] Said A., Laukkanen T., Järvinen M. (2016). Pilot-scale experimental work on
carbon dioxide sequestration using steelmaking slag. Appl. Energy.

[15] Jo H., Lee M. G., Park J., Jung K. D. (2017). Preparation of high-purity
nano-CaCO_3_ from steel slag. Energy.

[16] Niu Y.-Q., Liu J.-H., Aymonier C., Fermani S., Kralj D. (2022). Calcium carbonate: Controlled
synthesis, surface functionalization,
and nanostructured materials. Chem. Soc. Rev..

[17] Kato M., Hari T., Saito S., Shibukawa M. (2014). Determination
of free lime in steelmaking slags by use of ethylene glycol extraction/ICP-AES
and thermogravimetry. Tetsu-to-Hagané.

[18] Uehara N., Tanaka A. (2014). Change in characteristics
of ethylene glycol during
dissolving calcium oxide and its suppression. Tetsu-to-Hagané.

[19] Tanabe K., Ugomori A., Tohji K. (2010). Formation
process, morphology and
dispersibility of calcium carbonate in ethylene glycol solution. J. Soc. Inorg. Mater. Japan.

[20] Sasaki T., Ijima K., Takahashi Y., Murakami K., Sakai K., Kinoshita S., Toyama T. (2023). Extraction of calcium ions from steelmaking
slag with glycerol aqueous solution for calcium carbonate synthesis. J. Soc. Inorg. Mater. Japan.

[21] Sasaki T., Sakai K., Toyama T. (2025). Two-step precipitation synthesis
of CaCO_3_ from steelmaking slag in glycerol solution. ISIJ Int..

[22] Sasaki T., Suzuki A., Toyama T. (2025). Synthesis
of calcium carbonate using
calcium hydroxide from glycerol aqueous solution. J. Soc. Inorg. Mater. Japan.

[23] Callam C. S., Singer S. J., Lowary T. L., Hadad C. M. (2001). Computational analysis
of the potential energy surfaces of glycerol in the gas and aqueous
phases: Effects of level of theory, basis set, and solvation on strongly
intramolecularly hydrogen-bonded systems. J.
Am. Chem. Soc..

[24] Chelli R., Procacci P., Cardini G., Califano S. (1999). Glycerol condensed
phases Part II.A molecular dynamics study of the conformational structure
and hydrogen bonding. Phys. Chem. Chem. Phys..

[25] Dashnau J. L., Nucci N. V., Sharp K. A., Vanderkooi J. M. (2006). Hydrogen
bonding and the cryoprotective properties of glycerol/water mixtures. J. Phys. Chem. B.

[26] Marcus Y. (2000). Some thermodynamic
and structural aspects of mixtures of glycerol with water. Phys. Chem. Chem. Phys..

[27] Parsons M.
T., Westh P., Davies J. V., Trandum C., To E. C. H., Chiang W. M., Yee E. G. M., Koga Y. (2001). A thermodynamic study
of 1-propanol–glycerol–H_2_O at 25°C:
Effect of glycerol on molecular organization of H_2_O. J. Solution Chem..

[28] To E. C. H., Davies J. V., Tucker M., Westh P., Trandum C., Suh K. S. H., Koga Y. (1999). Excess chemical potentials, excess
partial molar enthalpies, entropies, volumes, and isobaric thermal
expansivities of aqueous glycerol at 25°C. J. Solution Chem..

[29] Hayashi Y., Puzenko A., Balin I., Ryabov Y. E., Feldman Y. (2005). Relaxation
dynamics in glycerol-water mixtures. 2. Mesoscopic feature in water
rich mixtures. J. Phys. Chem. B.

[30] Puzenko A., Hayashi Y., Ryabov Y. E., Balin I., Feldman Y., Kaatze U., Behrends R. (2005). Relaxation dynamics in glycerol-water
mixtures: I. Glycerol-rich mixtures. J. Phys.
Chem. B.

[31] Döss A., Paluch M., Sillescu H., Hinze G. (2002). From strong to fragile
glass formers: Secondary relaxation in polyalcohols. Phys. Rev. Lett..

[32] Fabri D., Williams M. A. K., Halstead T. K. (2005). Water T_2_ relaxation in
sugar solutions. Carbohydr. Res..

[33] Mizuno K., Miyashita Y., Shindo Y., Ogawa H. (1995). NMR and FT-IR studies
of hydrogen bonds in ethanol-water mixtures. J. Phys. Chem..

[34] Vanderkooi J. M., Dashnau J. L., Zelent B. (2005). Temperature
excursion infrared (TEIR)
spectroscopy used to study hydrogen bonding between water and biomolecules. Biochim. Biophys. Acta Gen. Subj..

[35] Zelent B., Nucci N. V., Vanderkooi J. M. (2004). Liquid
and ice water and glycerol/water
glasses compared by infrared spectroscopy from 295 to 12 K. J. Phys. Chem. A.

[36] Mendelovici E., Frost R. L., Kloprogge T. (2000). Cryogenic Raman spectroscopy of glycerol. J. Raman Spectrosc..

[37] Owczarek E., Hawlicka E. (2006). Molecular dynamics
study of CaCl_2_ in methanol. J. Phys.
Chem. B.

[38] Owczarek E., Rybicki M., Hawlicka E. (2007). Solvation
of calcium ions in methanol-water
mixtures: Molecular dynamics simulation. J.
Phys. Chem. B.

[39] Ren G., Ha Y., Liu Y.-S., Feng X., Zhang N. (2020). Deciphering
the solvent effect for the solvation structure of Ca^2+^ in
polar molecular liquids. J. Phys. Chem. B.

[40] Yang F., Liu Y.-S., Feng X., Qian K., Kao L., Ha Y. (2020). Probing calcium solvation by XAS, MD and DFT
calculations. RSC Adv..

[41] Fortnum D. H., Battaglia C. J., Cohen S. R., Edwards J. O. (1960). The kinetics of
the oxidation of halide ions by monosubstituted peroxides. J. Am. Chem. Soc..

[42] Annable T., Buscall R., Ettelaie R., Whittlestone D. (1993). The rheology
of solutions of associating polymers: comparison of experimental behavior
with transient network theory. J. Rheol..

[43] Sukenaga S., Ogawa M., Yanaba Y., Ando M., Shibata H. (2020). Viscosity
of Na–Si–O–N–F melts: Mixing effect of
oxygen, nitrogen, and fluorine. ISIJ Int..

